# The Sub-Cellular Localization of WRAP53 Has Prognostic Impact in Breast Cancer

**DOI:** 10.1371/journal.pone.0139965

**Published:** 2015-10-13

**Authors:** Laxmi Silwal-Pandit, Hege Russnes, Elin Borgen, Veronica Skarpeteig, Hans Kristian Moen Vollan, Ellen Schlichting, Rolf Kåresen, Bjørn Naume, Anne-Lise Børresen-Dale, Marianne Farnebo, Anita Langerød

**Affiliations:** 1 Department of Cancer Genetics, Institute for Cancer Research, Division of Cancer Medicine, Surgery and Transplantation, Radiumhospitalet-Oslo University Hospital, 0424 Oslo, Norway; 2 Department of Pathology, Division of Diagnostics and Intervention, Oslo University Hospital, 0450 Oslo, Norway; 3 Department of Oncology, Division of Cancer, Surgery and Transplantation, Oslo University Hospital, 0450 Oslo, Norway; 4 K.G. Jebsen Centre for Breast Cancer Research, Institute for Clinical Medicine, Faculty of Medicine, University of Oslo, 0313 Oslo, Norway; 5 Department of Breast- and Endocrine Surgery, Division of Surgery, Cancer and Transplantation, Oslo University Hospital, 0450 Oslo, Norway; 6 Department of Oncology-Pathology, Cancer Center Karolinska (CCK), Karolinska Institutet, SE-17176 Stockholm, Sweden; University of North Carolina School of Medicine, UNITED STATES

## Abstract

WRAP53 protein controls intracellular trafficking of DNA repair proteins, the telomerase enzyme, and splicing factors. Functional loss of the protein has been linked to carcinogenesis, premature aging and neurodegeneration. The aim of this study was to investigate the prognostic significance of WRAP53 protein expression in breast cancer. A tissue microarray was constructed from primary breast tumors and immunostained by a polyclonal WRAP53 antibody to assess the protein expression pattern. Two different patient cohorts with long term follow-up were studied; a test- and a validation set of 154 and 668 breast tumor samples respectively. Breast cancer patients with tumor cells lacking the expression of WRAP53 in the nucleus had a significantly poorer outcome compared to patients with tumor cells expressing this protein in the nuclei (HR = 1.95, 95%CI = 1.09–3.51, p = 0.025). Nuclear localization of WRAP53 was further shown to be an independent marker of prognosis in multivariate analysis (HR = 2.57, 95%CI = 1.27–5.19, p = 0.008), and also significantly associated with better outcome in patients with *TP53* mutation. Here we show that the sub-cellular localization of the WRAP53 protein has a significant impact on breast cancer survival, and thus has a potential as a clinical marker in diagnostics and treatment.

## Introduction

The WRAP53 protein is encoded by the *WRAP53* gene, which has a genomic location in head-to-head arrangement with the *TP53* gene on 17p13.1[1]. The protein WRAP53 (alias WRAP53β TCAB1 and WDR79) is 75kDa, consists of 548 amino acids and contains a highly conserved WD40 repeat domain. WRAP53 facilitates interactions of factors involved in splicing and telomere elongation and their assembly in nuclear organelles termed Cajal bodies[2–4].Depletion of WRAP53 interferes with the localization of telomerase in Cajal bodies and disrupts telomerase-telomere association, interfering with telomere synthesis and resulting in telomere shortening[2]. In addition, WRAP53 is an essential component of Cajal bodies and loss of this protein, or its aberrant overexpression, leads to collapse of these organelles and mislocalization of associated factors[3,4]. Recent findings also demonstrate that the WRAP53 protein is a regulator of DNA double-strand break repair by providing a scaffold for DNA repair proteins[5].

WRAP53 was first implicated in cancer in an extensive re-sequence analysis of *TP53* and its flanking genes, where single nucleotide polymorphisms (SNPs; rs2287498 and rs2287499; in linkage disequilibrium) was suggested to be associated with increased risk for developing Estrogen Receptor (ER) negative breast carcinomas[6]. The SNPs were further shown to be associated with elevated risk of developing aggressive ovarian cancer[7,8]. Moreover, inherited mutations in the *WRAP53* gene, resulting in impaired function of WRAP53, cause the cancer predisposition disorder dyskeratosis congenita[9,10]. A role of WRAP53 in cancer is further supported by observations of elevated levels of the protein in human cancer cells compared to normal cells[11,12]. WRAP53 knockdown through RNA interference was shown to trigger apoptosis in cancer cells but not in normal human fibroblasts, implying that WRAP53 expression is important for cancer cell survival[13]. Downregulation of WRAP53 in ovarian cancer was recently shown to correlate with defective DNA repair and poor clinical outcome ([14], *in press*). Similarly, loss of nuclear WRAP53 associates with poor prognosis in head and neck cancer[15].

Here, we have analyzed WRAP53 protein expression in breast carcinomas and show that the sub-cellular localization of the protein has an impact on the mortality of breast cancer patients.

## Material and Methods

### Patient material

The study cohort consists of 154 out of 212 primary breast tumor samples sequentially collected at Ullevål University Hospital, Norway from 1990 to 1994[16] (termed “test set”). A series of 668 from a total of 920 breast tumor samples collected in the Oslo Micrometastasis Study between 1995 and 1998 was used for validation[17] (termed “validation set”). The inclusion criterion was availability of formalin fixed paraffin embedded tumor tissue. The last clinical update of test set and validation set was performed in 2006 and 2010 respectively, providing an observation time of more than 10 years. All patients were treated according to Norwegian national guidelines at the time of diagnosis[16,17]. Written informed consent has been obtained from all the patients involved, and approval to perform studies on the patient material has been obtained from the Regional committee for medical research ethics, Norway.

### Tissue microarray construction

A tissue microarray (TMA) was constructed from formalin fixed and paraffin embedded primary breast tumor tissue (test set, n = 154) and consisted of three 0.6 mm paraffin cores from each individual tumor (selecting the most aggressive components and minimum of non infiltrating components), that altogether constituted five recipient paraffin blocks. TMA for the validation set (n = 668) was constructed following the similar procedure, but with two cores from each individual tumor that altogether constituted eight recipient paraffin blocks.

### Immunohistochemistry (IHC) analysis

The TMA sections were immunostained by a rabbit polyclonal WRAP53 antibody (referred to as WRAP53-C1; Innovagen AB, Sweden), targeted against amino acids 483–496 of full length WRAP53 protein. The antibody specifically detects WRAP53 protein in cells as affirmed by western blot analysis [1]. The deparaffinized and rehydrated tissue array sections of the test set were subjected to Heat Induced Epitope Retreival (HIER) using 10 mMCitrate Buffer (pH6) and microwave oven prior to immunostaining. Polymer based IHC was done using Dako EnVision^+^ System–HRP (DAB) kit (Dako, Glostrup, Denmark) and Dako Autostainer following manufacturer’s standard procedure. Following treatment with a peroxidase block, the TMA sections were immunostained with WRAP53-C1 primary antibodies (diluted 1:1000) and subsequently with secondary antibody; peroxidase-labelled polymer conjugated to goat anti-rabbit immunoglobulins. HIER procedure for the validation set was performed in a water bath based Dako PTLink instrument using EnVision^TM^ FLEX Target Retrieval solution, Low pH (corresponds to citrate-buffer pH6). The sections were stained with WRAP53 antibody (diluted 1:500) using Dako EnVision^TM^ Flex+ system. For signal amplification, the sections were further incubated with EnVision^TM^ Flex+ rabbit LINKER. The immunostained sections (both test and validation set) were then visualized with 3’3 diaminobenzidine tetrachloride (DAB), a chromogenic reporter DAB, counterstained with hematoxylin, dehydrated and cover-slipped with Richard-Allan Scientific Cytoseal XYL (Thermo Scientific, Waltham, MA, USA).

### IHC scoring

The stained sections in both the test- and validation set were visually inspected and scored in random order for the WRAP53 protein expression using light microscopy. The criteria for a sample to be scorable were set to the presence of at least one core containing at least 50 intact tumor cells. Some internal background was noticed for several samples and if the intensity of such cytoplasmatic staining was above the level of staining in other cell types it was scored as positive. WRAP53 expression in nucleus and cytoplasm were scored separately and the samples were categorized based on the fraction of stained cells (Nucleus; 0: No tumor cells with distinct staining of the nucleus, 1: <5% of the cells stained, 2: 5–50% of the cells stained, 3: 50–75% of the cells stained, and 4: >75% of the cells stained; and, Cytoplasm; 0: No tumor cells with distinct staining of the cytoplasm, 1: faint staining of the cytoplasm or <5% of the cells stained, and 2: >5% of the cells stained). Samples with below 5% positively stained cells were considered negative for protein expression. For patients with discrepant results between the individual cores from the same tumor, the core with highest percentage of positively stained cells was taken into account. The semi quantitative scoring criteria were established by two pathologists, and cases with discrepant results were discussed to obtain consensus.

### Statistical Analysis

All statistical analysis were performed using R version 3.0 with the package ‘rms’[18]. Survival analysis was performed using the Kaplan-Meier estimator with log-rank test for significance, and Cox proportional hazard (PH) models were used for Hazard Ratio estimations. Pearson’s chi-squared (χ^2^) test and Fisher’s exact test were used when appropriate to test association between different variables.

## Results

### The WRAP53 protein is localized both in the cytoplasm and nucleus of breast tumor cells

**Fig 1:**
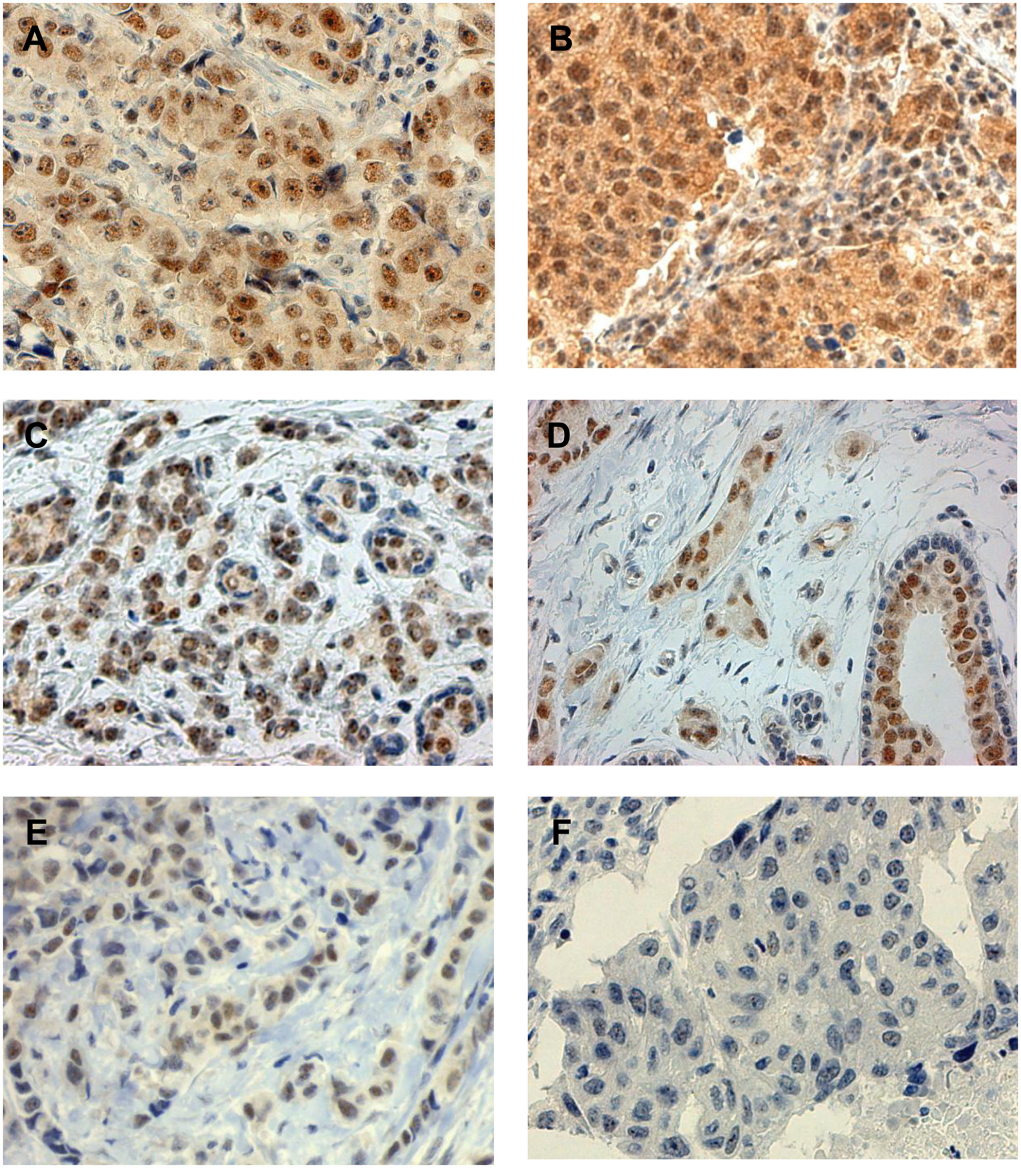
Immunohistochemical staining patterns of WRAP53 in breast cancer. **A.** and **B.** Positive nucleus/positive cytoplasma, **C.** and **D.** positive nucleus/negative cytoplasma (with normal mammary epithelial cells), **E.** positive nucleus/negative cytoplasm (invasive lobular carcinoma), and **F.** negative nucleus/negative cytoplasm.

**Fig 2:**
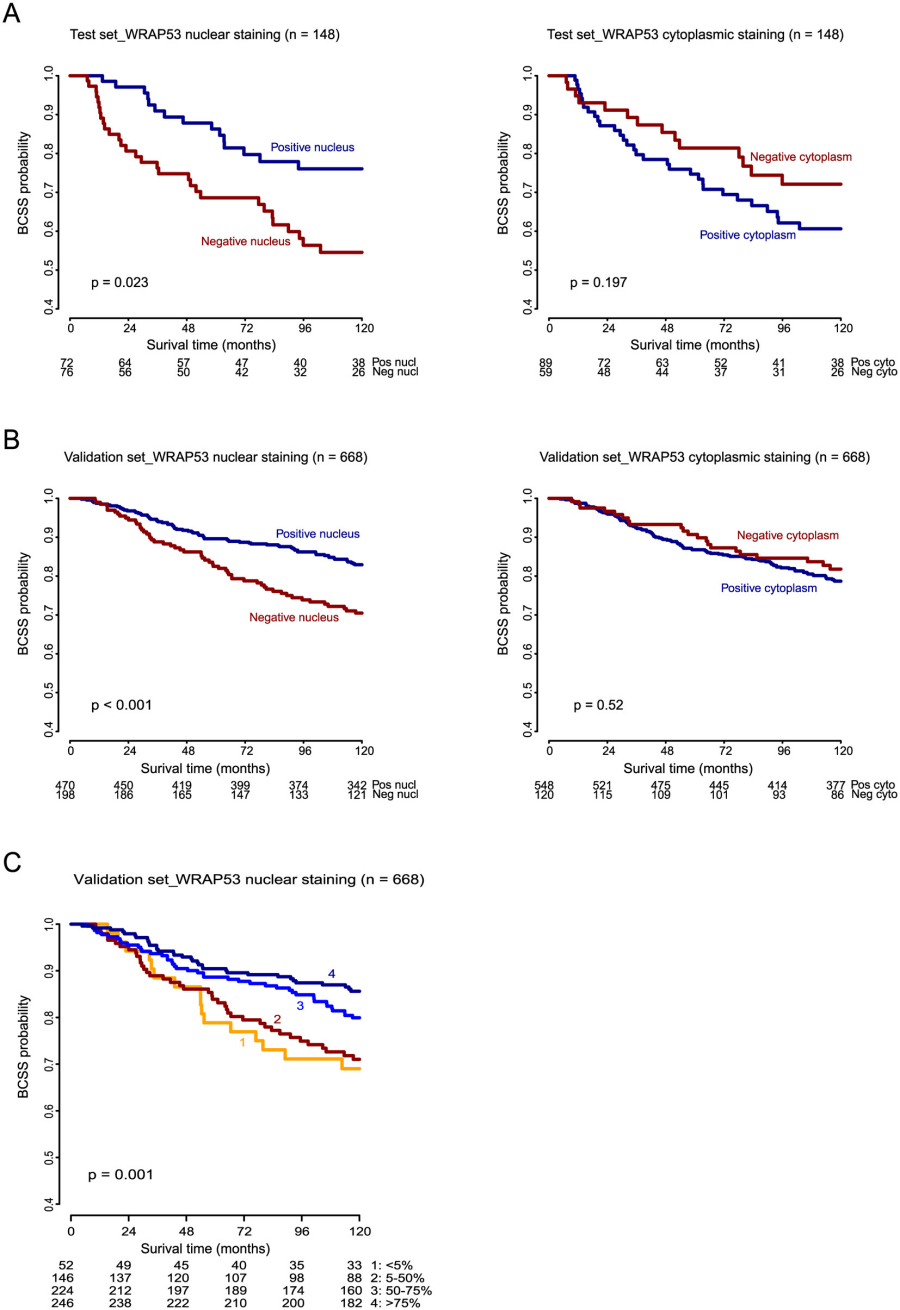
Kaplan-Meier curves showing Breast Cancer Specific Survival (BCSS) of WRAP53 IHC staining of nucleus and cytoplasm **A.** in the test set and **B.** in the validation set. **C.** BCSS of WRAP53 nuclear IHC staining in validation set categorized based on fraction of stained cells. 0: no tumor cells with distinct staining of the nucleus, 1: <5% of the cells stained, 2: 5–50% of the cells stained, 3: 50–75% of the cells stained, and 4: >75% of the cells stained. Numbers at risk are listed below each chart.

The WRAP53 protein was found to localize both in the nucleus and the cytoplasm of the breast carcinoma cells, separately or in combination (Fig 1, S1 Table and S2 Table). A substantial diversity in WRAP53 expression levels was observed across the breast tumor samples (Fig 2C). Expression of this protein was observed also in normal mammary epithelial cells, but to a lesser extent than in the majority of malignant breast tissue. In normal breast ducts it was primarily the inner luminal cells, not the surrounding myoepithelial cells that expressed the WRAP53 protein (Fig 1D). Sequencing of WRAP53 gene in the test set revealed that somatic mutations in the gene are not common events in breast cancer (data not shown). Hence, the staining pattern is not associated with the mutational status of the *WRAP53* gene.

### Association of the WRAP53 protein with clinical variables

In the test set, WRAP53 protein expression did not demonstrate a statistically significant association with any of the commonly used clinico-pathological and molecular markers (S3 Table). In the validation set, an association between WRAP53 and tumor size, node status and grade was observed (S4 Table).

### Prognostic impact of sub-cellular localization of WRAP53

Univariate survival analysis showed that breast cancer patients with negative nuclear staining of the WRAP53 protein had a statistically significantly increased mortality compared to patients with positive nuclear staining (HR = 1.95, 95%CI = 1.09–3.51, p = 0.025; Fig 2A, S5 Table). These results were validated in the larger series of 668 patients (HR = 1.83, 95%CI = 1.32–2.52, p < 0.001; Fig 2B, S5 Table). Furthermore, an increasing degree of nuclear staining was correlated with improved survival (validation set; p = 0.001, log-rank test; Fig 2C). Conversely, patients with positive staining of the protein in the cytoplasm tended to have a poorer clinical outcome in the test set, than those with negative staining in the cytoplasm, although statistical significance was not reached (Fig 2A and 2B).

**Fig 3:**
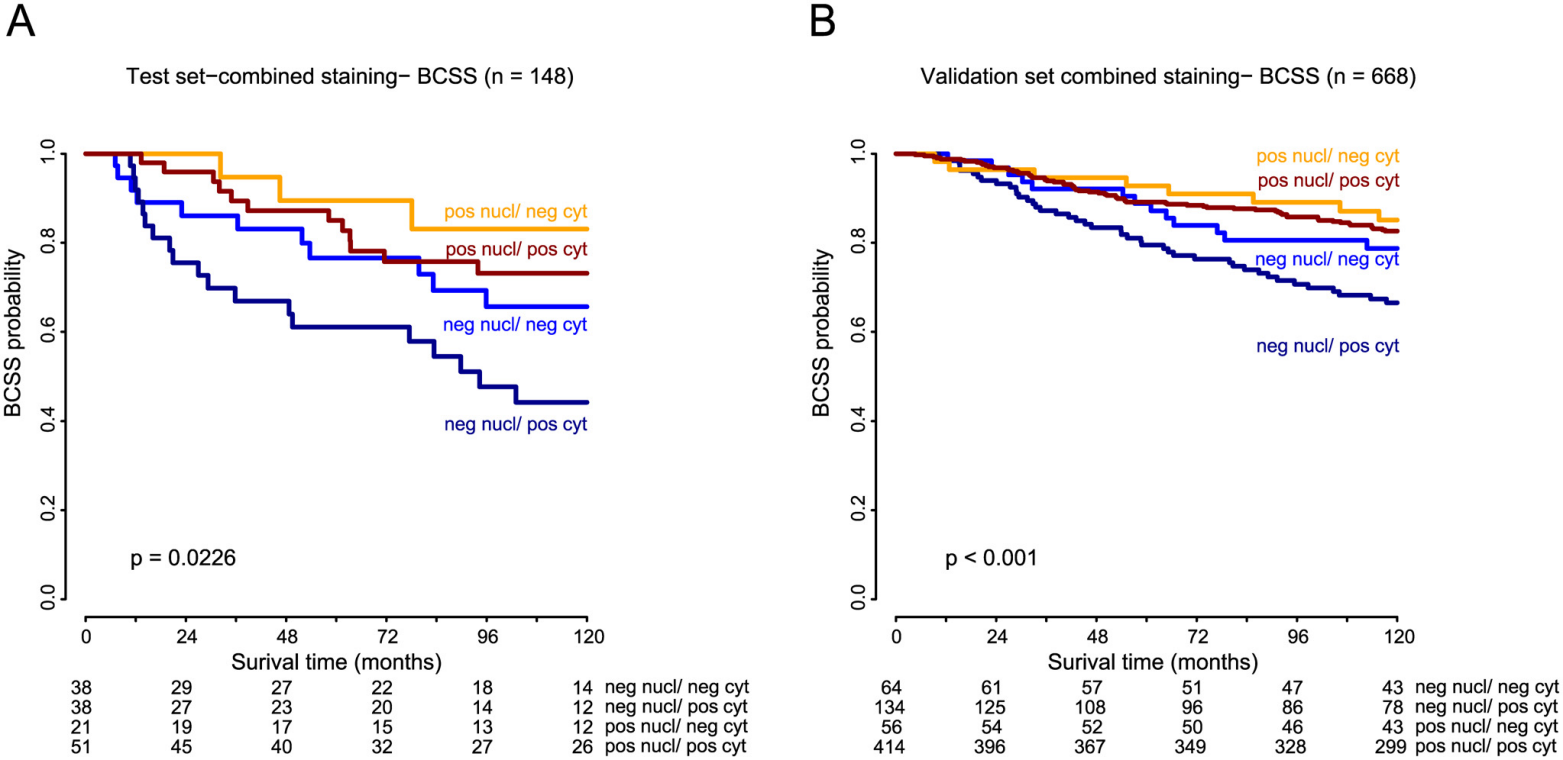
Kaplan-Meier curves showing Breast Cancer Specific Survival (BCSS) of WRAP53 IHC staining of nucleus and cytoplasm in combination, **A**. in the test set and **B.** in the validation set. Numbers at risk are listed below each chart.

When the combined effect of WRAP53 expression in the sub-cellular compartments on patient outcome was investigated, negative nuclear WRAP53 combined with positive cytoplasmic WRAP53 was associated with reduced survival (p = 0.023, log-rank test; Fig 3A). This observation was also confirmed in the validation set (p = 0.001, log-rank test; Fig 3B).

In multivariate analyses, WRAP53 nuclear staining was shown to be an independent marker of prognosis, both in the test set (HR = 2.57, 95%CI = 1.27–5.19, p = 0.008; Table 1) and the validation set (HR = 1.55, 95%CI = 1.10–2.18, p = 0.011; Table 1). WRAP53 demonstrated prognostic power also in a subset of patients not given systemic adjuvant treatment (validation set, p = 0.001, log-rank test).

**Table 1:** Multivariate Cox regression analysis (BCSS).

	Test set (n = 154)			Validation set (n = 668)		
	Multivariate Cox Regression analysis			Multivariate Cox Regression analysis		
Variables	Hazard Ratio (HR)	95% CI of HR	p-value	Hazard Ratio (HR)	95% CI of HR	p-value
Tumor size						
T1	1			1		
T2	1.62	1.10–4.64	0.269	1.46	1.03–2.12	**0.0420**
T3_T4	2.36	1.27–8.24	0.131	2.28	1.30–3.98	**0.0030**
Node status						
Node neg	1			1		
Node pos	2.92	1.42–6.00	**0.003**	2.69	1.87–3.88	**< 0.001**
Tumor grade^1^						
Grade 1 vs 2 vs 3	1.46	0.73–2.90	0.276	2.07	1.52–2.82	**< 0.001**
ER status						
Negative	1			1		
Positive	1.20	0.56–2.58	0.640	0.77	0.51–1.18	0.240
TP53 Mutation status						
Wildtype	1					
Mutated	3.78	1.71–8.37	**0.001**			
WRAP53 nuclear staining						
Positive	1			1		
Negative	2.57	1.27–5.19	**0.008**	1.55	1.10–2.18	**0.011**

^1^ Tumor grade is included as ordinal variablePrognostic impact of *WRAP53* in patients stratified for ER- and *TP53-*status

It is well recognized that ER positive and ER negative breast cancer are dissimilar diseases. Stratifying the test-set by ER status showed that positive nuclear staining of WRAP53 predicted outcome in patients with ER negative disease only (p = 0.016; S1A Fig). In the validation set the prognostic effect was statistically significant in the ER positive group (S1B Fig). The WRAP53α transcript is a natural antisense RNA of the *TP53* gene involved in posttranscriptional regulation of *TP53*. We therefore investigated the association between WRAP53 staining and breast cancer survival in *TP53* wildtype and *TP53* mutated samples separately. In the test-set we found that positive nuclear staining was predictive of outcome in patients with *TP53* mutation (p = 0.031; S1C Fig), but not in those with wild type *TP53*. In the validation set the prognostic effect of WRAP53 was statistically significant in both TP53 wild type and mutant tumors (S1D Fig).

## Discussion

Previously, the WRAP53 protein has been reported to be overexpressed in a broad range of cancer cell lines compared to non transformed cells[13], as well as in primary rectal tumor cells compared to normal mucosal cells[12]. Recently, increased expression of WRAP53 was observed in esophageal squamous cell carcinoma tissue compared to adjacent non-neoplastic esophageal mucosa tissue[11]. In agreement with these reports, we found that the WRAP53 protein in general showed higher expression in malignant breast tissue than in normal mammary epithelial cells. The observed differential expression of the protein between various malignant cells and normal cells suggests a role for the protein in cancer pathogenesis.

In this study, we describe the sub-cellular localization of WRAP53 in breast tumors and show that the protein expression impacts clinical outcome, independent of other common prognostic markers. Patients seemed to benefit from WRAP53 expressed in the nucleus by showing a better outcome of the disease. Also, increasing the degree of nuclear WRAP53 expression correlated to better clinical outcome (Fig 2C). The finding underscores the importance of the sub-cellular localization of the protein, which should be accounted for in prediction of outcome. A similar observation was reported in head and neck cancer, where loss of nuclear WRAP53 is associated with reduced survival and enhanced radioresistance in patients with head and neck cancer. This correlation was observed only for WRAP53 expression in the nucleus and not in the cytoplasm [15].

The role of WRAP53 in formation and maintenance of Cajal bodies is largely affected by its proper localization. Mahmoudi *et al*. showed that ectopic overexpression (very high expression) of WRAP53 prevented formation of Cajal bodies and resulted in more dispersed localization of the protein in the nucleoplasm instead of its normal accumulation in Cajal bodies[4]. This implies that the expression level of this protein does not necessarily indicate the presence of a functional protein. A more detailed characterization of the expression pattern is thus required to be able to accurately interpret the good outcome observed in patients with positive nuclear WRAP53 staining. In the context of breast cancer, the localization of the protein in cytoplasm seemed to be less important, as it was not significantly correlated to survival.

The WRAP53 protein has recently been shown to regulate the assembly of repair factors at sites of DNA damage. Consequently, loss of this protein was found to disturb repair of DNA double-strand break and resulted in the accumulation of spontaneous DNA breaks[5]. Moreover, WRAP53 has been identified in several proteomic and genome wide siRNA screens designed to detect factors associated with the DNA damage response[19–21]. Involvement of WRAP53 in DNA repair is also supported by the association of SNPs in WRAP53 with benzene-induced hematotoxicity that is linked with deficiency in DNA repair[22] and the finding that inherited WRAP53 mutations cause dyskeratosis congenita, characterized by deficit DNA repair machinery among others[23]. The association of reduced WRAP53 expression with worse clinical outcome potentially could be related to its role in DNA repair and the genomic instability that would follow the loss of this protein in the nucleus. Indeed, recent reports show that downregulation of WRAP53 in ovarian cancer leads to defective DNA repair and poor clinical outcome of the patients ([14], *in press*).

Aberrant localization of proteins may be caused by mutation, altered expression of cargo proteins or deregulated trafficking machinery, and contributes to the pathogenesis of many human diseases, such as cancer (reviewed in [24]). Consistent with the low WRAP53 mutation frequency reported in the Cosmic database[25], somatic mutations were rare in the breast cancer cohort studied here. Mutations therefore do not explain the localization pattern observed for WRAP53 in these tumors. Little is known about the regulatory machinery involved in WRAP53 transport between and within the cellular compartments, and is important to investigate because the function of WRAP53 seems to be governed by appropriate localization of the protein.

The association of WRAP53 with some clinicopathological variables (tumor size, node status and grade) was observed only in the validation set. Furthermore, our study does not define a patient group (based on ER status and *TP53* mutation status) that could have an increased clinical benefit of nuclear WRAP53. Nuclear WRAP53 predicted outcome in the ER negative subgroup and in the *TP53* mutated group in the test set. In the validation set, significant prognostic effect was observed in ER positive and in both*TP53* mutated and *TP53* wildtype group. These differences can partly be explained by the differences between the two cohorts, where the tumors in the test set overall can be characterized as more aggressive and advanced than those in the validation set. Also, the larger sample size of the validation cohort may have allowed discovering the subtle associations, which, in the test set, could not be discovered due to smaller patient cohort. Another important discrepancy is the prognostic effect of ER status and *TP53* mutation status in these two cohorts. While, ER-status is not prognostic in the test set, it is highly prognostic in the validation set, whereas the opposite is true for *TP53* mutation status. Thus far, there has not been reported any direct associations between TP53 and WRAP53 at the protein level. The sense-antisense posttranscriptional regulatory relationship between *TP53* mRNA and the overlapping isoform WRAP53α observed in cell-lines[1,26] could not be confirmed in the context of breast tumor tissue due to expression of WRAP53α mRNA below detection limits in the breast tumors (test set; Taqman RT-PCR; data not shown). Nevertheless, it is interesting that the WRAP53 protein predicted survival in the *TP53* mutated group in the test set, which is a poor prognosis group and lacks robust prognosis markers; the finding calls for further validation.

This study is the first to describe the sub-cellular localization of the WRAP53 protein in primary breast carcinomas and identify WRAP53 as a potential prognostic biomarker. We show here that the prognostic value of this protein in breast cancer is dependent on its sub-cellular localization, where the presence of WRAP53 in the nucleus correlates to better patient outcome. The prognostic impact of nuclear WRAP53 in patients with *TP53* mutation should be further explored to elucidate its potential as a new biomarker for predicting outcome in this poor prognosis group.

## Supporting Information

S1 Fig Kaplan-Meier curves showing BCSS of WRAP53 IHC staining of nucleus; stratified for 2 ER status, **A**. in the test set and **B.** in the validation set, and stratified for TP53 mutation status, 3 **C.** in the test set and **D.** in the validation set. Numbers at risk are listed below each chart.
(EPS)

S1 Table Immunohistochemical staining data of the test set. (XLSX)

S2 Table Immunohistochemical staining data of the validation set. (XLSX)

S3 Table Association of WRAP53 nuclear staining with clinical and histopathological characteristics in the test set. (XLSX)

S4 Table Association of WRAP53 nuclear staining with clinical and histopathological characteristics in the validation set. (XLSX)

S5 Table Univariate Cox regression analysis (Breast Cancer Specific Survival) on the clinical and histopathological variables in the test and validation set. (XLSX)
